# Acceptance and adjustment: A qualitative study of experiences of hearing and vision impairments and daily life among oldest old recipients of home care

**DOI:** 10.1111/opn.12236

**Published:** 2019-05-17

**Authors:** Gro Gade Haanes, Elisabeth O.C. Hall, Grethe Eilertsen

**Affiliations:** ^1^ Faculty of Natural and Health Sciences, Department of Nursing University of the Faroe Islands Tórshavn Faroe Islands; ^2^ Section of Nursing, Department of Public Health Aarhus University Aarhus Denmark; ^3^ Faculty of Health and Social Sciences, Department of Nursing and Health Sciences University of South‐Eastern Norway Drammen Norway

**Keywords:** daily life, hearing impairment, home care, oldest old, qualitative research, vision impairment

## Abstract

**Introduction:**

The severity of age‐related hearing and vision impairments increases with age. Such sensory impairments are risk factors for functional decline, reduced social participation, withdrawal, depression and accidents, and hence they make people vulnerable and adversely affect their quality of life.

**Aim:**

The aim of this study was to explore how the oldest old recipients of home care experience sensory impairments in daily life.

**Design:**

An inductive, descriptive research design was used.

**Method:**

Ten recipients of home care with a mean age of 89 years were interviewed in their homes. The study was implemented in accordance with the suggestions from Elo and Kyngäs for inductive content analysis.

**Findings:**

The main theme concerned acceptance and adjustment in daily life. Subcategories concerning the category of reduced hearing were identified as “acceptable though annoying” and “hesitant about using hearing aids.” Subcategories concerning the category of reduced vision were “reading is increasingly challenging” and “living with vision diseases.” The third category of feeling weak was elucidated in the subcategories “troublesome bodily changes” and “strenuous days with limited energy.”

**Conclusions:**

It is imperative to recognise that the oldest old are in a distinct phase of the lifespan. Despite this population being aware of their hearing and vision impairments, they do not always have the strength to alter the situation. Instead they accept it; they often struggle with more serious health challenges. Therefore, they are not prioritising using their limited energy reserves to try to improve or optimise their hearing and vision impairments themselves.

**Implications for practice:**

The oldest old with sensory impairments cannot be expected to perform all the necessary activities of daily living or address their functional sensory impairments. Close monitoring and assistance need to be applied to the oldest old.

**What does this research add to existing knowledge in gerontology?**

- The oldest old typically face several serious health challenges that limit their capacity to prioritise hearing and vision impairments.
- The oldest old are characterised by feeling weak and having few energy reserves.
- Troublesome bodily changes together with sensory impairments make the oldest old vulnerable and at risk of being socially isolated.
- Many of the oldest old have limited possibilities due to their poor health condition to get out of their homes in order to receive specialised treatment for their hearing and vision impairments.

**What are the implications of this new knowledge for nursing care with older people?**

- We suggest engaging the oldest old in energy conservation techniques and addressing their sensory impairments through education of the benefits of intervention to preserve as much hearing and vision as possible using compensatory techniques.
- To prevent feeling of social isolation, it is important to offer the oldest old assistance for improving their hearing and vision functions.
- We suggest that home care nurses and other community healthcare providers increase their attention towards informing the oldest old clients about age‐related hearing and vision impairments and possible interventions to improve these functions.
- We suggest home care nurses and other community healthcare providers to increase their awareness towards possible consequences of sensory impairments in everyday matters for the oldest old; such as telephone calls, changing light bulbs and transportation.
- The oldest old and the providers of home care should be offered knowledge in how to optimise the indoor lighting conditions and its meaning for vision and hearing.

**How could the findings be used to influence policy or practice or research or education?**

- The oldest old need help to improve their hearing and vision function in order to increase the probability of them maintaining social contact with others.
- We suggest establishing interdisciplinary teams involving nurses, audiologists, low‐vision occupational therapists and optometrists that can visit older people in their homes to provide advice on how to alleviate changes in hearing and vision function.
- Attention is needed on the importance of the senses to maintaining social contact and activity among the oldest old.
- Preventive teams in municipalities or home care services should start monitoring hearing and vision from an earlier age.
- More research is needed to establish individualised interventions to help the oldest old to preserve and improve their hearing and vision.

## 1 INTRODUCTION

The increase in life expectancy is resulting in more people experiencing hearing and vision impairments. Even the fact that there is close relationship between ageing and sensory impairment, people affected would not always understand this (Wallhagen & Strawbridge, 2017). In particular the oldest old would also, in many instances, suffer from decreasing health overall. Hence they require special attention from healthcare services. Nurses and other healthcare providers may facilitate both social and other activities for old people with dual sensory impairments. Though, in order to deliver optimum support, it is crucial to understand how old people suffering from these impairments experience this, and how they cope in daily life.

## 2 BACKGROUND

Age‐related changes in hearing and vision mean that most people will experience increasingly impaired hearing and vision function as they grow older (Cacchione, 2014; Wyller, 2011). Age‐related hearing impairment (presbyacusis) is characterised by reduced hearing sensitivity and speech understanding in noisy environments, and an impaired ability to localise sound sources, mostly because of hair cells in the cochlea are lost with advancing age. Furthermore, many old people are troubled by the increased production of cerumen in the outer ear canal, which may prevent sound from reaching the eardrum (Wyller, 2011).

Age‐related changes in the vision system (presbyopia) mean altered or impaired vision. The pupil becomes smaller, which reduces the amount of light entering the eye, the lens thickens, gets stiffer, and is less able to accommodate, and the ciliary muscle weakens (Wyller, 2011). In addition, eye diseases such as age‐related macular degeneration (AMD), diabetic retinopathy, senile cataract and glaucoma are multifactorial conditions that involve complex interactions among various physiological processes (Grue, 2010).

Several studies have documented that hearing and vision impairments (i.e., dual sensory impairments) are common among people older than 80 years (Chia et al., 2006; Saunders & Echt, 2007). The prevalence reportedly varies from 25% to 78% among those aged 80 years and older, and is notably lower in studies based on self‐reporting (Caban, Lee, Gómez‐Marin, Lam, & Zheng, 2005; Hannaford et al., 2005). Likewise reported is that this age group gradually adapts to worsening hearing and vision; they do not always notice it (Lin, 2012; Cacchione, 2014; Haanes, Kirkevold, Horgen, Hofoss, & Eilertsen, 2014). Serious hearing and vision impairments might also be misinterpreted as failing intellect (Cacchione, 2014), cognitive decline (Lin et al., 2013; Pacala & Yueh, 2012; Wallhagen & Strawbridge, 2017), delirium (Cacchione, Culp, Dyck, & Laing, 2003; Inouye, Westendorp, & Saczynski, 2013), functional decline (Cacchione, 2011; Li et al., 2011; Wood et al., 2011) and falls (Cacchione, 2010; Dillion, Gu, Hoffman, & Ko, 2010; Lord & Dayhew, 2001; Lopez et al., 2011). Further, several studies report hearing and vision loss to be associated with depression (Brody et al., 2001; Cacchione, 2011; Crews & Campbell, 2004; Li et al., 2011) and negative impact on the quality of life (Chia et al., 2006; Wallhagen & Pettengill, 2008). However, people in this age group are reportedly hesitant to seek help about their sensory impairments, especially regarding hearing (Solheim, Kværner, Sandvik, & Falkenberg, 2012; Haanes, 2016).

Haanes, Kirkevold, Hofoss, and Eilertsen (2015) showed that hearing and vision impairments negatively affect self‐care abilities among the oldest old recipients of home care and that simply providing information and advice are not sufficient for managing the impairments. Studies have revealed that a significant number of hearing‐impaired older persons find it difficult using hearing aids (Solheim et al., 2012; Haanes, 2016; Pronk et al., 2017), and they have fewer positive expectations and are more problem‐oriented toward using hearing aids (Solheim, Kværner, & Falkenberg, 2011).

In a previous study, we examined sensory impairments among people aged 80+ years who received home care and found that their self‐assessments of hearing and vision were weakly correlated with the results obtained using standardised instruments (Haanes et al., 2014). We subsequently performed an exploratory randomised, controlled trial of hearing and vision impairments in the same sample; 100 participants were randomised into the intervention group (IG) and the control group (CG). Participants were screened for functional hearing and vision and lighting conditions in their home. The intervention lasted for 10 weeks; it included advice and self‐care support in relation to hearing, vision and indoor lighting conditions, and IG participants were given individual advice. These advices comprised the use of hearing aids, removal of earwax, custom‐made spectacles and increasing the indoor lighting levels (for more information see Haanes et al., 2015, Haanes, 2016). We found that self‐assessed hearing and vision significantly improved in the IG group (Haanes et al., 2015). These findings indicated the need for a deeper exploration of how the oldest old themselves describe their experience living with sensory impairments, and thus form the background of the present qualitative study.

## 3 AIM

The aim of this study was to explore how the oldest old recipients of home care experience sensory impairments in daily life.

## 4 DESIGN AND METHOD

We used an explorative design with in‐depth individual interviews. Qualitative interviews are designed to gain knowledge about experiences of participants in relation to a particular phenomenon (Malterud, 2001), which in the present study was living with impaired hearing and vision in old age.

### 4.1 Participants, sampling and setting

After the end of the intervention study mentioned above, we invited 14 participants from the IG to an interview study (Table 1). Fatigue and poor health resulted in that four of them declined to participate. The final sample thus comprised ten participants (eight women and two men). The interviews provided rich and varied descriptions, and so we considered the sample appropriate.

**Table 1:** Inclusion and exclusion criteria

Inclusion criteria	Exclusion criteria
≥80 years old	Presence of dementia^a^
Receiving home care	Cognitive impairment^a^
Included in the intervention group	Very serious illness^a^
Able to speak Norwegian	
Signed written informed consent	

a Assessed by the nurses who recruited the participants.

The mean age of the participants was 89 years. All were living at home (in four rural areas and one small town) in the southern Norway. Age and different diseases meant that all participants received public home care ranging from once a week to several times a day (Table 2). Seven lived in detached houses and three lived in apartments. Eight lived alone and two lived with a spouse. Specialist treatment for hearing and vision was available within a 1‐hr drive, and there were opticians and audio pedagogues in each municipality.

**Table 2:** Characteristics of the study sample (*n* = 10)

Participant number	Age/gender	Living situation	Hearing impairment measured by Pure Tone Average (PTAV)^a^	Vision impairment measured by Visual Acuity (VA)^b^	Other health impairments revealed during the interviews	Home care frequency
1	89/male	Lives with spouse	Moderate impairment	Visually impaired	Age‐related macular degeneration (AMD), prostate cancer	Once a week
2	91/female	Lives alone	Moderate impairment	Slightly visually impairment	Dizziness, balance problems, has fallen several times	Once a week
3	90/female	Lives alone	Moderate impairment	Slightly visually impairment	Heart disease, uses pacemaker, back pain, problems with walking	Once a day
4	87/female	Lives with spouse	Moderate impairment	Slightly visual impairment	Heart and kidney failure, weakened force in right arm	3 times a day
5	88/female	Lives alone	Moderate impairment	Visually impaired	Eye infection after eye operation	Once a week
6	92/female	Lives alone	Moderate impairment	Slightly visually impairment	Recovering after fall and pneumonia	Once a week
7	90/female	Lives alone	Moderate impairment	Slightly visually impairment	Pain in the back (bed in living room)	Once a week
8	91/male	Lives alone	Moderate impairment	Slightly visually impairment	Cataract, AMD, needs help with ADL	2–3 times a day
9	85/female	Lives alone	Moderate impairment	Slightly visually impairment	Amputation of both legs, sits in wheelchair, needs help with ADL	2–3 times a day
10	86/female	Lives alone	Moderate impairment	Slightly visually impaired	Lung disease, uses O_2_‐flask at home	Once a week

a Degree of hearing loss according to WHO reference values (World Health Organization, 2014). No impairment or very slight impairment measured by pure tone audiometry (PTAV). Frequencies measured are 500 Hz, 1000 Hz, 2000 Hz and 4000 Hz. PTAV is the average measurement of these four frequencies. No impairment or very slight hearing problem PTAV < 25 dB, light impairment (hearing aid may be needed) PTAV 25–40 dB, moderate impairment (hearing aid usually recommended) PTAV 40–60 dB, severe hearing loss (hearing aid needed/lip reading) 60–80 dB, profound PTAV > 80 dB.

b Degree of vision impairment according to WHO reference values (WHO, 2014), measured by visual acuity on a LogMAR chart. Normal vision VA > 0.8, slightly visually impaired 0.4–0.8, visually impaired VA < 0.4.

### 4.2 Data collection

One in‐depth interview was conducted with each participant in their homes by the first author. The interviews focused on experiences with hearing and vision impairments in daily life. To encourage the participants to freely express themselves, the interviewer adopted an open discussion approach (Elo & Kyngäs, 2008). After providing introduction to the study, the first question asked was “Please tell me about your hearing and hearing challenges.” Similar questions were asked about vision experiences. In two of the interviews, the spouse of the participant was present in the room: in one case, this was the husband of a female participant, and in the other, it was the wife of a male participant. In both cases, the spouses had not been invited to participate, and they occasionally interjected during the interview, which sometimes included correcting what the interviewed person said. The interviews were digitally recorded and lasted from 30 to 60 min.

### 4.3 Ethics

The information letter used in the intervention study contained information about a subsequent interview study. Participants were informed that they could be contacted and asked to participate. Written informed consent was obtained from all participants. The phrasing on the information sheet and consent forms was easy to read, that is we used bold letters and font 14. Nurses responsible for recruitment were instructed to read the information out loud for the participants if needed. Moreover, the participants assured the nurses that information given was properly understood. The participants were also orally informed about the purpose and methodology and that they had the right to withdraw from the study without consequences. Approval was granted by the Regional Committees for Medical and Health Research Ethics (approval number 2011/1408) and by the Norwegian Social Science Data Service (reference number 28025).

### 4.4 Data analysis

The interviews were transcribed verbatim into a 134‐page document. Following the suggestions for inductive content analysis (Elo & Kyngäs, 2008), the process was performed in phases. First, the authors read the full text to get an overview of the data. Second, we organised the content by grouping meaning units about hearing and vision. Since the participants also spoke about other health issues and everyday life, data about these issues were similarly grouped. Third, grouped data (words and meaning units) that shared the same meaning were clustered into categories and subcategories. The analysis was more circular than linear throughout this process. To consider trustworthiness, credibility, dependability and confirmability (Polit & Beck, 2017), all of the authors checked the elaborated findings for relevance while carefully considering the meaning units and clustering before finalising the categorisation and a main theme.

## 5 FINDINGS

Nearly all participants were aware of their reduced hearing and vision. They felt weakened and were also coping with other health challenges with the result that these impairments were not prioritised. The main theme therefore was found to be *acceptance and adjustment in daily living*. Reduced hearing was accepted but annoying, especially when accompanied by other conditions. Hearing aids were sometimes considered a nuisance, and the participants were therefore hesitant to use them. Reduced vision was accepted but challenging, especially when reading. Daily life seemed to be more a matter of accepting bodily changes, somatic diseases and limited energy in general than the limited social life caused by their dual sensory impairment. Below we elucidate the main theme through categories and subcategories. For pragmatic reasons, we present the two impairments separately, even though all of the participants had dual sensory impairment.

### 5.1 Reduced hearing

#### 5.1.1 Acceptable though annoying

Most participants described their hearing as acceptable though creating problems in many daily situations. One woman in her 90s stated: “On Monday I was at a birthday party and I did hear, but again and again I had to ask the ones who spoke to repeat. I could not really follow the conversation.” This indicated that reduced hearing made the participants feel excluded. The hearing impairment was especially annoying during social events: “The worst thing is when many are present…. I must try to keep up the spirit. There is nothing else to do.”

Some participants stated that when more than one person was talking, it sounded like incomprehensible buzzing: “With 30 persons in a room, and you are talking to the one you're with, then you can't hear everything. No one can.” This might cause them to withdraw, and some stated that they seldom went to social meetings: “I always feel left out since I can't be part of the conversation.”

However, some participants considered their hearing to be good enough. For them, the matter was to “…hear the TV, the telephone and the doorbell.” They were not concerned about asking someone to repeat a comment**:** “If somebody speaks to me and I do not immediately catch it, I just reply ‘what’.”

Some participants simply accepted their hearing impairment, others did not openly admit it, even though they often asked for repetition and clarification during the interview. When asked, one of them stated: “My hearing is good,” while another described that her son disagreed: “I think my hearing is not that bad, but my son thinks it is.”

Many found attending check‐ups with audiologists away from home tiresome and draining of their strength and resources. Even regular hospital visits were not always followed up: “It is difficult to visit a hearing specialist and I should have been at the hospital to control my pacemaker.”

#### 5.1.2 Hesitant about using hearing aids

For various reasons, most participants were hesitant about using hearing aids. Some had previously tried without success, and some had heard rumours that using hearing aids was a struggle; they did not want to bother with it. One stated: “I have a neighbor who is using hearing aids in both ears. It is a real ordeal. She would get rid of them if she could.” Furthermore, one participant could not lift her arm to place and remove the aid, and she needed help to handle the batteries: “That arm will not function anymore; I can't lift it further than this high.”

### 5.2 Reduced vision

#### 5.2.1 Reading is increasingly challenging

The participants enjoyed reading, especially the daily newspaper. However, they worried about the small letters and found it difficult to read newspaper print. One stated: “When I am going to read the newspaper, I can't read it with these bifocal glasses. I have been thinking of doing something about it.” They considered newspapers to be important even though they did not read much of the content. One said: “I only read the headlines.... I can't manage to read the articles.” It seemed that they thought it useless to improve their vision. “My poor vision emerged gradually. I was operated for cataract and my vision functioned well for a while. Now it's worse. There are things I don't see.”

#### 5.2.2 Living with vision diseases

Some participants had for years received treatment for serious vision diseases such as glaucoma or AMD. One of them experienced the onset of vision deterioration over a single day and mentioned that the ophthalmologist had not detected the underlying disease:

The optician noticed something wrong with my right eye. I was sent to the hospital eye clinic. There the doctor thought my vision was good. But shortly after, almost overnight, it changed. So I was sent to the hospital again. Then it was too late to save my vision.

Another participant suffering from AMD complained that her vision sometimes appeared foggy. She first experienced the problem when perceiving that her bathroom tiles were uneven. The doctor told her that nothing could be done. Her husband, who was present at the interview, then interjected that his wife had received laser treatment for the calcifications in AMD, which had improved her vision somewhat. Another participant experienced deteriorated vision:

It crept onto me without me really noticing. I honestly didn’t know. I had a cataract operation, and after that I really felt that my eyesight improved. Now, it has deteriorated to worse than before that operation.

Living with serious chronic vision diseases were part of the daily life, though being thankful for help and having a positive attitude were prominent. One woman reported that she had very good eyesight, but had to use eye drops. The home care nurse who came four times every day helped her to administer eye drops and eye ointment. However, for her this was a trifle, she had a good life overall. She was happy that going to bed safely every night.

### 5.3 Feeling weakened—strenuous days with limited energy

Even though living with hearing and vision impairments was originally the main focus of the study, the participants spontaneously provided information about other health and illness challenges that occupied their minds. They were particularly concerned about pain, slowness, tiredness, forgetfulness and living alone. Such matters were sometimes quite a struggle for them. We interpreted this as a feeling of being weakened marked by strenuous days with limited energy.

#### 5.3.1 Troublesome bodily changes

Several of the participants suffered from severe health challenges such as heart and kidney failure. They also talked about their painful body, aching hips and shoulders, stiff knees or degenerated back. One 90‐year‐old woman stated that “My back has degenerated beyond repair. This cannot be reversed, he [the doctor] said.” These bodily changes were in the forefront of their minds**.** Their daily life had become strenuous, and they had to economise their physical and mental capacity. From this perspective, it seemed as they considered the sensory impairments as secondary. One stated:

Yes, because I have heart and kidney failure, they have to tuck me in, and use an ambulance take me to visit a hearing or vision specialist. I cannot stand on my own feet anymore

#### 5.3.2 Strenuous days with limited energy

The participants admitted having limited energy in different ways and that their days unfolded slowly. Everything was time‐consuming, with one of them saying: “I spend a very long time doing everything I do.” They needed to take rests during the day, and their tiredness was conspicuous: “Oh yes, ask me if I'm tired”!

The gradual deterioration in their ability to think clearly and their increasing forgetfulness greatly concerned several of the participants. Some of them worked on developing strategies to manage these changes, such as putting things in fixed places and systematically writing things down that they needed to remember. All of them had similar experiences:

More often than not, when I try to recollect something it is completely empty. Nothing comes to mind. I am terrible when it comes to remembering things. If I do not leave things in a certain place I would almost never recall where I left them.

Their weak and troublesome bodies had significantly reduced how far they were able to move or travel. This resulted in them spending most of the time indoors, particularly during the winter months: “I have not been outside quite often. During the winter, I am inside all the time.” Many of the participants spent much of their time watching TV.

Despite the weakness and limitations that they described, they hesitated to ask neighbours or the providers of home care for small services. Sometimes, changing a light bulb or repairing tiny things in the house could become a difficult situation for them:

One of the neighbors who is helping me bought a lightbulb for me for the hallway, but the lightbulb he bought is very dim. Therefore, it is always very dark in there. That is wearing me out. Sometimes I have asked the home care nurse to change some of the lightbulbs for me.

The participants did not want to be of bother to others, and so mostly they had to manage everything on their own and felt that they did not receive much help in their daily lives. Their days were strenuous and they had few energy reserves.

## 6 DISCUSSION

In this study, we report findings based on interviews with ten oldest old people with hearing and vision impairments. The participants were living at home (most of them alone), and they all received public home care at least weekly. This study was designed to explore how the oldest old manage their hearing and vision impairments when living at home and receiving home care. The interviews revealed that hearing and vision impairments formed an integral part of their living experience. They considered hearing and vision impairments to belong to the image of being very old; it was something they accepted having to live with even though it was sometimes quite annoying.

Reduced hearing was particularly troublesome when they were with many other people at social gatherings; it had the effect of impairing their social life. Reduced vision was annoying when it made reading the newspaper difficult or when they had to ask for help with changing light bulbs or other simple practical everyday tasks. Such findings concur with previous reports on how hearing and vision impairments affect everyday competencies (Tiwana, Benbow, & Kingston, 2016; Bjørnsdottir, 2017). Hearing impairment in particular is a burden in social life, while vision impairment strongly impacts everyday competencies (Heyl & Wahl, 2014). Thus, both hearing and vision impairments markedly influence the social and daily lives of the oldest old.

Our finding that the participants were hesitant about using hearing aids confirms previous reports (McCormack & Fortnum, 2013; Hickson, Meyer, Lovelock, Lampert, & Khan, 2014; Ng & Loke, 2015) that large percentages of older people who would benefit from hearing aids do not use them. Kochkin (2000) stated that the hearing aid “stays in the drawer.” The older old participants in our and other studies particularly consider background noise, lack of perceived benefit, and problems with fit and comfort to be reasons for their nonuse (Kochkin, 2000; Hickson et al., 2014). Even though hearing‐aid technology has advanced from analog to digital signal processing, such challenges remain prevalent, which makes significant others and also professionals important players not only in communicating about hearing aids in general, but also in preparing for and providing daily assistance in their use (Ng, 2015). The participants in our study needed help to place their hearing aids properly. It might be that the home care workers were not aware of the problems experienced by the oldest old in using hearing aids, they might have been unable or not allowed to assist, or they were not assigned to help with this task. We would argue that inadequate knowledge and practical skills among home care workers may have serious consequences for the hearing problems experienced by the oldest old.

Previous research has revealed that only a minority of staff at nursing homes receive training on the use and care of hearing aids, and that a correspondingly low proportion believe they have sufficient knowledge about the hearing aids used by residents, including about how to change hearing‐aid batteries (Solheim, Shiryaeva, & Kværner, 2016). We think that such a lack of training and knowledge might also be prevalent among home care professionals.

Reading daily newspapers was another challenging problem for the oldest old in this study. It was clear that our participants were interested in following current events. The oldest old belong to a generation that is used to reading, and especially reading newspapers in paper form. Therefore, not being able to read the news because of vision impairment could remind them that they themselves are “outdated.” They might read their newspaper for pleasure and leisure, to keep up with what was happening and for learning and being independent. Difficulty reading regular print has previously been described as a threat to leisure time and well‐being in later life (Ryan & Bernard, 2003). Magnus and Vik (2016) found that reading, among groups of Canadian and Norwegian old people, was just as important after as it was before they experienced reduced vision.

Our findings similarly concur with findings from a meta‐synthesis exploring well‐being and adjustment to vision loss late in life (Nyman, Dibb, Victor, & Gosney, 2012). That meta‐synthesis revealed that many people with vision impairment or vision loss late in life suffer from not being able to frequent the cinema, drive, travel or read. Several of the participants in our study lived with serious chronic vision diseases, which is common for people in their 80s and 90s (Wyller, 2011). Even when people who have an age‐related eye diagnosis are receiving treatment, the visual function gradually deteriorates, often without them noticing. Careful monitoring of the vision is therefore of the utmost importance in this age group. Like the participants in the study of Nyman et al. (2012), those in our study learned to live with their vision impairment. Some accepted it, while others more or less denied it or refused to accept it; they kept asking themselves “Why me”?

We interpreted the oldest old as being weakened. Even after their participation in the intervention study (in which they had improved their self‐assessed hearing and vision significantly), their ability to socialise with others was limited by both bodily and sensory challenges. Their physical decline resulted in them spending most of the time indoors. They were tired, had few energy reserves and their hearing and vision impairments were barriers in communicating with others as well as being informed about current events. Altogether, our understanding was that they seemed to consider that nothing could be done about their impairments. The oldest old in our study demonstrated a wish to take care of themselves as much as possible, not wanting to bother others with daily trifles. Although adjustment was not always easy, they expressed feelings of gratitude for living a long life and for feeling safe and still being able to manage in everyday life. However, they constantly had to struggle with health issues and weakness from being old; their strength and capability to perform even small tasks was decreased, which meant that their sensory impairments were not prioritised. These findings concur with the model of Rowe and Fried (2013) of how resilience turns to frailty in old age. That model highlights that even the most resilient person is frail when reaching a very old age and increases the dependence even for performing simple daily activities. Research into living late in life (Hinck, 2004; Ness, Hellzen, & Enmarker, 2014; Grøn, 2016) has documented that oldest old people are frail, vulnerable, and suffering from losses and chronic health conditions, and the frailty and vulnerability seem to coexist. Inner strength is needed for the oldest old to accept life transitions, sickness, losses and loneliness (Lang, Michel, & Zekry, 2009; Nygren, Norberg, & Lundman, 2007). We would argue that rather than the factors coexisting, there is an oscillation between bothering, accepting, adjusting and agency. Our arguments coincide with Pusztai (2015, p. 329) describing that living in old age involves carefully walking across the tightrope of

learning new ways of being and doing, changes of the “I am” and “I am not” and the “I can” and “I cannot,” all point to ways of adapting and coping with the challenges that old age has wrought on bodies, minds, abilities, and life circumstances, it is the attitude, inner negotiations, will‐power, and habits of positivity and gratitude, that prevent the I cannot and the I am not from leading to depression or despair.

Good lighting is important for both hearing‐impaired and vision‐impaired people, so that they can see and read the face and mouth of the person they are talking to. Since advancing age results in less light entering into the eye, it makes one more dependent on good lighting conditions to see well. Eilertsen, Horgen, Kvikstad, and Falkenberg (2016) found that even though the lighting levels in the homes of healthy home‐dwelling 75‐year‐old Norwegians were worryingly low, they expressed that the lighting levels were good. The present study did not explicitly focus on this topic, but taking the results from that previous study into account, we recommend that both older people themselves and their healthcare providers in the primary healthcare system pay attention to optimising the indoor lighting conditions of the oldest old in order to ensure healthy ageing in the home environment.

Høy, Wagner, and Hall (2007) described self‐care as a health resource among older people, and this also applies to the oldest old who were addressed by the present study. Self‐care though is a broad concept (Matarese, Lommi, Marinis, & Riegel, 2018) closely linked to symptom management and symptom management support. The oldest old, in taking care of themselves, need assistance and support, rather than acceptance from healthcare providers, in managing their sensory impairments and everyday lives**.** Small things really matter. We would argue that providers of care in the home should be more proactive about solving even the tiniest problem, since the oldest old, as part of their self‐care, need help in setting goals during their often‐strenuous daily lives. At the same time, it is challenging for these care providers to understand the unique situation of the care receiver. This study has revealed that the oldest old who need home care because of age‐related declines and diseases have reduced energy reserves. Thus, they need to carefully consider how to spend their limited energy. The oldest old used their strength in accepting and adjusting to the most urgent issues in their daily lives and therefore decided that their hearing and vision were acceptable. They had to take this approach, and so it might not be realistic to expect them to use their limited energy reserves to try to improve or optimise their hearing and vision themselves. Accordingly, we suggest establishing interdisciplinary teams that include nurses, audiologists, optometrists, low‐vision occupational therapists and other professionals to visit older people in their homes and advise them on how to alleviate their hearing and vision impairments. Cacchione (2014) emphasises the importance of nurses to collaborate with their colleagues to investigate nursing interventions in identifying and treating sensory impairments in older adults to improve their quality of life.

### 6.1 Strengths and limitations of the study, and future recommendations

In this interview study, we explored experiences of the oldest old and generated knowledge about how they feel forced to prioritise their resources to manage more serious health challenges than their sensory impairments. Nevertheless, we were not sufficiently aware of how weak the participants were when planning for the interviews and subsequently found that most of the participants were easily tired and could not manage long conversations. Therefore, transferring the present findings to other settings or groups of oldest old should be done with caution. Two spouses were present in two of the interviews, which according to Norlyk, Haahr, and Hall (2015) might have influenced the results. Researchers should consider such methodological issues in future studies of the oldest old. It might be better to arrange repeated and shorter interviews and be prepared for the spouse to be present. A major strength of this study was its focus on the oldest old. This distinct phase of the lifespan calls for knowledge considering the experiences and perspectives of very old people in order to optimise the development of home care for them. Further research is needed to increase knowledge of whether screening and implementing interventions at an earlier age might better preserve hearing and vision and thus prevent social isolation and improve quality of life among the oldest old.

7

**Implications for practice**

- We suggest engaging the oldest old in energy conservation techniques and adressing their sensory impairments through education of the benefits of intervention to preserve as much hearing and vision as possible using compensatory techniques.
- To prevent feeling of social isolation, it is important to offer assistance for improving their hearing and vision functions. Home care nurses and other health care providers should increase their attention towards informing the oldest olds about age‐related hearing and vision impairments and possible interventions to improve these functions.
- Home care nurses and other health care providers should increase their awareness towards possible consequences of sensory impairments in everyday matters for the oldest old, such as transportation, telephone calls and changing light bulbs.
- Oldest old and providers of home care should be offered knowledge in how to optimise the indoor lighting conditions and its meaning for hearing and vision.

## 8 CONCLUSION

The oldest old are in a distinct phase of the lifespan, and home care providers can offer support and care in a variety of meaningful ways. Even when the oldest old are aware of their hearing and vision impairments, they do not always have the strength to improve the situation. Instead, they accept it, often struggling with more serious health challenges, unable to prioritise their limited energy for hearing and vision impairments. We encourage policymakers to facilitate hearing and vision monitoring at an early age and to obtain the information necessary to detect age‐related changes in functioning of the senses. When people know about normal age development of their senses early in the lifespan, they can start the self‐monitoring process that helps improve their hearing and vision function in old age.

## CONFLICT OF INTEREST

No conflict of interest. All authors have confirmed agreement with the final statement of the study.

## AUTHOR CONTRIBUTION

Study design: GGH, GE; Data collection: GGH; Data analysis: GGH, EH, GE; Manuscript preparation: GGH, EH, GE.
